# A cost analysis of a cancer genetic service model in the UK

**DOI:** 10.1007/s12687-016-0266-4

**Published:** 2016-02-27

**Authors:** Ingrid Slade, Helen Hanson, Angela George, Kelly Kohut, Ann Strydom, Sarah Wordsworth, Nazneen Rahman, for the MCG programme

**Affiliations:** 1 Oxford University Hospitals NHS Trust, Oxford, UK; 2 Health Economics Research Centre, Nuffield Department of Population Health, University of Oxford, Old Road Campus, Headington, Oxford, OX3 7LF UK; 3 The Ethox Centre, University of Oxford, Oxford, UK; 4 The Division of Genetics and Epidemiology, The Institute of Cancer Research, London, UK; 5 Cancer Genetics Unit, Royal Marsden NHS Foundation Trust, London, UK; 6 TGLclinical, The Institute of Cancer Research, London, UK

**Keywords:** Costs, Cancer genetics, Genetic services, BRCA, Breast cancer

## Abstract

Technological advances in DNA sequencing have made gene testing fast and more affordable. Evidence of effectiveness and cost-effectiveness of genetic service models is essential for the successful translation of sequencing improvements for patient benefit, but remain sparse in the genetics literature. In particular, there is a lack of detailed cost data related to genetic services. A detailed micro-costing of 28 possible pathways relating to breast and/or ovarian cancer and BRCA testing was carried out by defining service activities and establishing associated costs. These data were combined with patient-level data from a Royal Marsden Cancer Genetics Service audit over a 6-month period during which BRCA testing was offered to individuals at ≥10 % risk of having a mutation, in line with current NICE guidance. The average cost across all patient pathways was £2227.39 (range £376.51 to £13,553.10). The average cost per pathway for an affected person was £1897.75 compared to £2410.53 for an unaffected person. Of the women seen in the Cancer Genetics Service during the audit, 38 % were affected with breast and/or ovarian cancer, and 62 % were unaffected but concerned about their family history. The most efficient service strategy is to identify unaffected relatives from an affected individual with an identified BRCA mutation. Implementation of this strategy would require more comprehensive testing of all eligible cancer patients, which could be achieved by integrating BRCA testing into oncology services. Such integration would be also more time-efficient and deliver greater equity of access to BRCA testing than the standard service model.

**Electronic supplementary material:**

The online version of this article (doi:10.1007/s12687-016-0266-4) contains supplementary material, which is available to authorized users.

## Introduction

Health care policy initiatives in recent years in the United Kingdom (UK) and elsewhere have recommended that healthcare services integrate advances in genomic technologies and knowledge into clinical practice for the benefit of patients (“Our Inheritance, Our Future. Realising the potential of genetics in the NHS.” 2003; “Genomic Medicine” 2009; Brand and Lal 2012; “Realising the potential of stratified medicine” 2013). The technological advances are increasingly enabling gene testing to be offered via multigene panel, exome or whole genome testing which potentially allows greater throughput of samples and significant economies of scale (Shendure et al. 2004). However, the decision-making process regarding the affordability of the expansion and integration of genomic technologies into health services is impeded by limited clarity of where costs lie in the current standard genetic service model(s) and where genomic testing could best fit into these models.

Clinical cancer genetic units offer services to individuals and families with the goal of assisting treatment decisions in patients with a cancer diagnosis and facilitating early cancer detection and cancer prevention for any future cancers for them and their relatives. Mutations of over 100 genes are known to cause an increased risk of cancer, and these underlie approximately 3 % of cancer overall (Rahman 2014; Vencken et al. 2013). There is strong evidence that identification of cancer predisposition gene mutations has an impact on diagnosis and management of cancer patients and their families (Rahman 2014; Vencken et al. 2013; Byrski et al. 2012; Turner and Tutt 2012; Fong et al. 2009).

A large proportion of the work of any cancer genetic service is the management of familial breast and ovarian cancer. Germline mutations in *BRCA1* and *BRCA2* (collectively termed ‘BRCA’) underlie a proportion of both these cancers, and the most recent guidelines for familial breast cancer in the UK published in 2013 recommend testing individuals at ≥10 % chance of having a mutation (NICE 2013).

Typically in the UK, and many other countries, patients requiring assessment for cancer gene testing are referred to a cancer genetic service, where application of risk thresholds, such as the 10 % threshold for BRCA testing, recommended by the National Institute for Health and Care Excellence (NICE) is used to manage resource allocation (NICE 2013). However, it has been demonstrated that not all those eligible for testing are being referred to cancer genetic services. For example, ∼15 % of high-grade serous ovarian cancer is due to germline BRCA mutations and thus are eligible for testing at a 10 % risk threshold (Alsop et al. 2012; Zhang et al. 2011). However, referral of ovarian cancer patients to genetic services is very low, around 7–20 % (Alsop et al. 2012; Zhang et al. 2011; Fong et al. 2009). In part, this is because only about half of mutation-positive ovarian cancer patients report a significant family history of cancer (Alsop et al. 2012; Zhang et al. 2011). Clearly, important opportunities for improved management of ovarian cancer patients and cancer prevention in their relatives are being missed through the existing processes. Moreover, the cancer genetic service delivery model has limited staff numbers and an infrastructure that is not easily adapted to accommodate unmet need, or to address the increasing demand for cancer gene testing (Fong et al. 2009).

Evidence of effectiveness and cost-effectiveness of service models, including the use of genomic technologies, is essential for policy-making frameworks but remains sparse in the genetics and genomics literature (Sullivan et al. 2012). In particular, there is lack of cost data, including readily available published reference costs, for genetic services. Micro-costing is a detailed costing approach and requires identification, measurement and valuation of all underlying activities of a service (Gray et al. 2011). In this paper, we present a full micro-costing, from the healthcare provider perspective, of a cancer genetic service for breast and ovarian cancer within the UK National Healthcare Service (NHS) from referral to management.

Our study was undertaken as part of the Mainstreaming Cancer Genetics (MCG) programme (www.mcgprogramme.com), a translational initiative that is developing the assays, informatics, clinical infrastructure, education and evaluation to allow implementation of cancer gene testing into routine clinical care of cancer patients and their relatives.

## Methods

To perform the micro-costing, we first mapped out all the possible pathways relating to breast and/or ovarian cancer and BRCA testing that a patient may follow when referred to the Royal Marsden Cancer Genetics Service prior to the implementation of mainstream testing in June 2013. We believe these to be generally representative of most cancer genetic services in the UK in 2014. Once completed, the service activities and resources involved in each step of every pathway were defined and the costs for each activity established so that the overall cost of each patient pathway could be calculated based on 2013 costs.

### Pathway costings

**Fig. 1:**
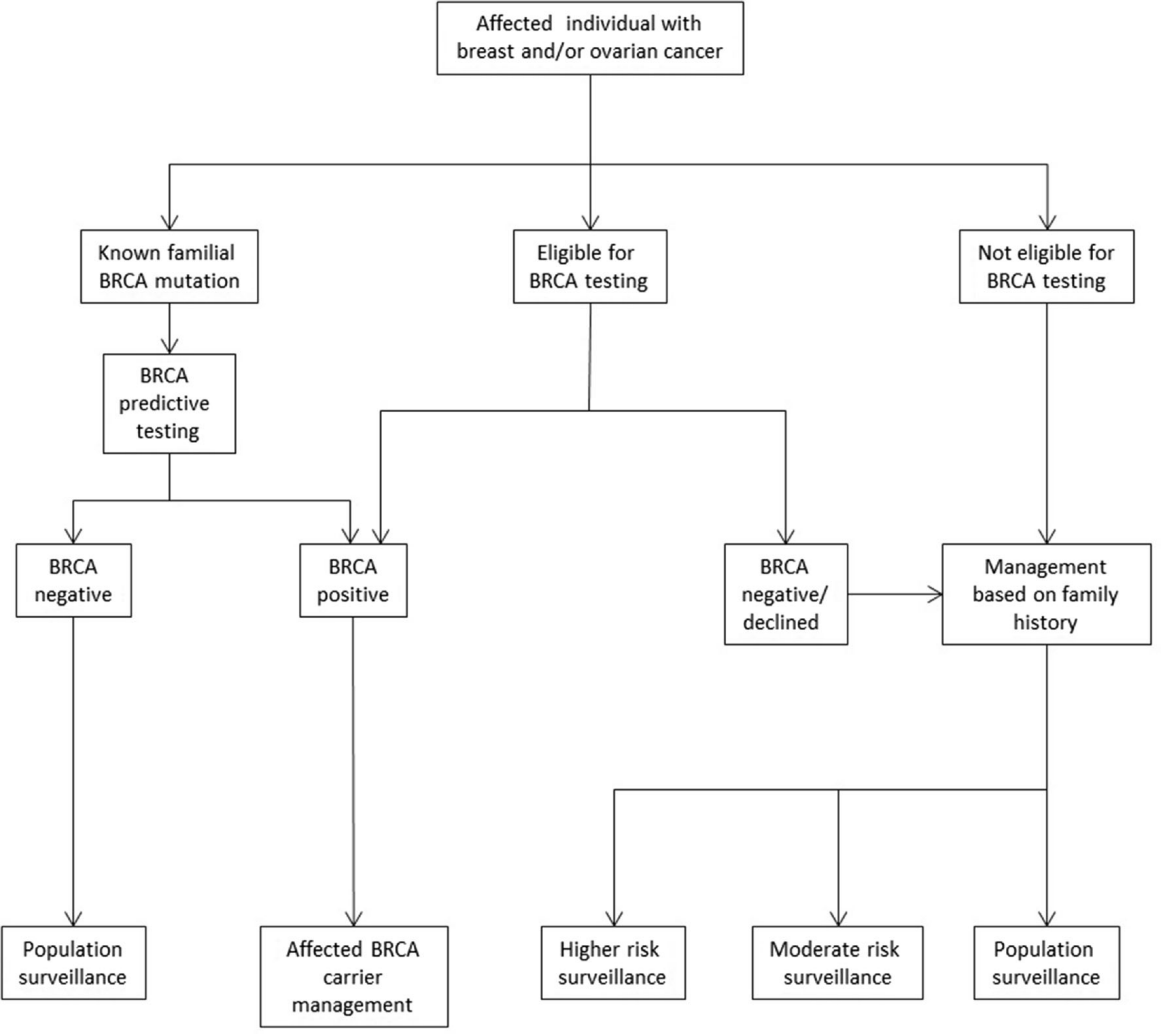
Individual affected with breast or ovarian cancer-patient pathways

**Fig. 2:**
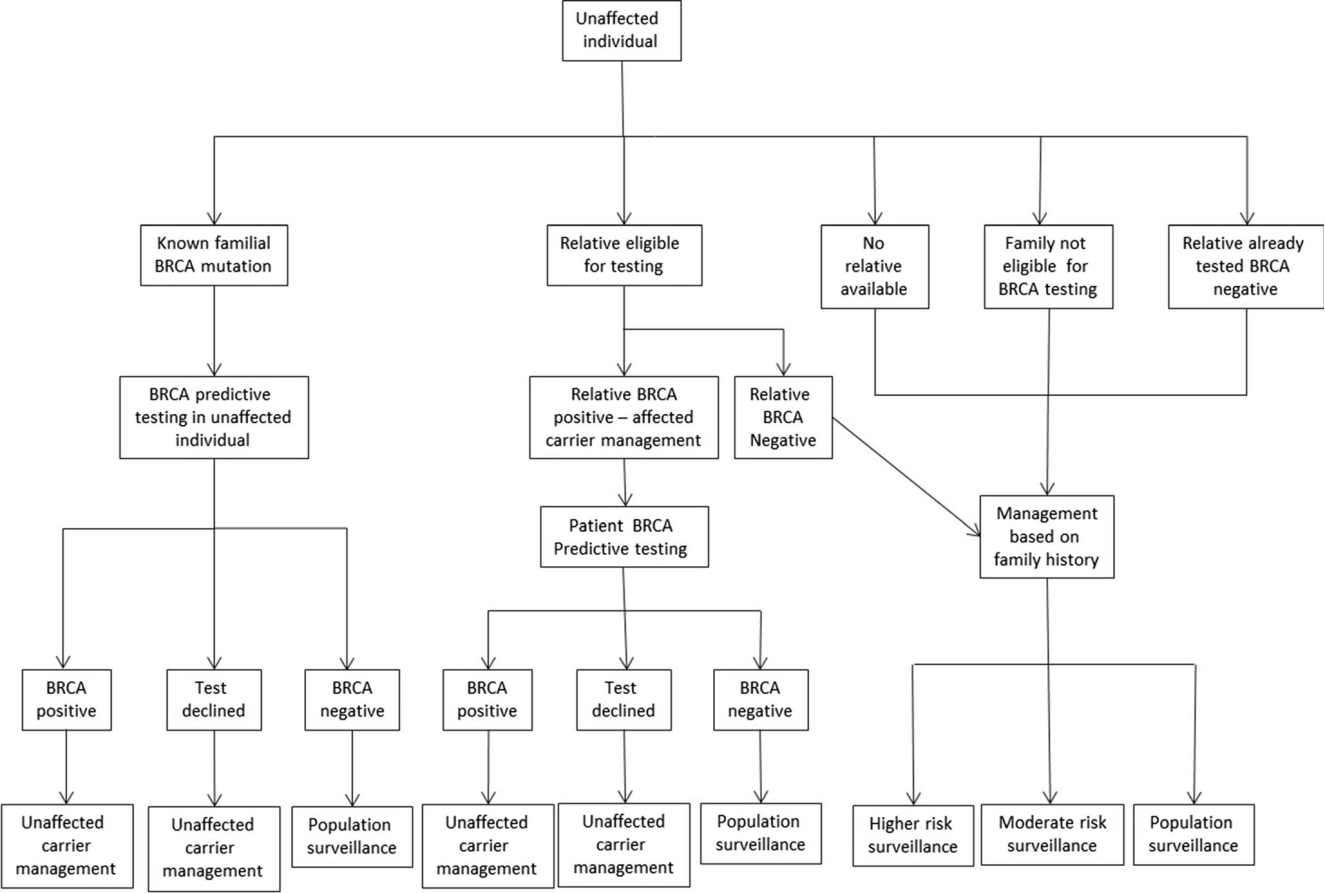
Individual unaffected-patient pathways

The patient pathways were defined from referral to surveillance management using service protocols and in discussion with the Cancer Genetics Unit at the Royal Marsden NHS Foundation Trust in London (Figs. 1 and 2). The management strategy for each patient was as described in the Royal Marsden Cancer Genetics management protocols in use during the audit period (Supplementary Figure 1). These are based on offering testing to those with ≥10 % risk threshold of having a BRCA mutation, in line with the current NICE guidance (NICE 2013). If a BRCA mutation is not identified, surveillance recommendations are made according to the individual’s family history. Population surveillance recommendation was costed for mammography 3 yearly from 50 to 70 years of age; moderate risk surveillance is annual mammography from 40 to 50 years of age and then entering the population surveillance; higher risk surveillance is annual mammography 40 to 50 years 18 monthly mammograms from 50 to 60 years and then entering the population surveillance programme as per RMH protocols (Supplementary Figure 2) (2011).

In order to maintain a manageable number of patient pathways, in families with no BRCA mutation, it was assumed that relatives were in the same risk group as the proband. In families where an individual with a BRCA mutation is identified, relatives either carry the mutation (or chose not to be tested) with subsequent surveillance management as mutation carriers, or they do not carry the familial mutation and are managed with population-level surveillance through the NHS. For individuals where a BRCA mutation is identified, carrier surveillance comprises annual mammography 40 to 70 years and annual MRI 30 to 50 years. In addition, mutation carriers are eligible for risk-reducing surgery, bilateral mastectomy and/or bilateral salpingo-oophorectomy. The uptake of these interventions in unaffected carriers was determined using expert opinion from the clinical unit alongside literature estimates, 30 % for bilateral mastectomy and 60 % for bilateral salpingo-oophorectomy (Metcalfe et al. 2008; Meijers-Heijboer et al. 2003; Evans et al. 2009). For affected carriers, the 5-year survival rate at age 40 years (0.7 for breast cancer and 0.69 for ovarian cancer www.ons.gov.uk) was incorporated. It was assumed that 5 % of ovarian cancer patients with a BRCA mutation that survive to 5 years would undergo bilateral mastectomy. These rates were based on discussions with the Royal Marsden Cancer Genetics Unit, as no published data were available.

In order to identify resource use in each pathway clinical, administrative and laboratory staff were asked to estimate the length of time each defined activity took them in minutes, the general quantity of consumables and where each activity took place in the pathway. The full BRCA test includes comprehensive analysis of the full coding sequence and intron-exon boundaries for small intragenic mutations and larger exonic deletion/duplications. A predictive test describes a targeted analysis for a specific mutation already known to predispose to cancer in the family. For this paper, we used the TGLclinical Sanger sequencing + MLPA sequencing cost for full gene testing, which was charged to the Royal Marsden NHS Foundation Trust in 2013 exclusive of VAT costs as per NICE guidance. It should be noted that there is variability in BRCA gene test costs across the NHS, and the TGLclinical test cost was at the lower end of the range. The NICE guidance used a comparable test cost of £700 which is reflected in the sensitivity analysis (NICE 2013). Additionally, the testing is now performed with NGS technology using the TruSight Cancer Panel (see www.TGLclinical.com for further details). The cost of post genetic testing management, which includes mammography, MRI, mastectomy and salpingo-oopherectomy, was taken from 2013 NHS reference costs (NHS reference costs 2012) which are published average costs derived from hospital trust submissions and the literature (Taylor-Phillips et al. 2012; NICE 2013) (Table 1).

**Table 1:** Unit costs for clinical activity and patient management costs (2013)

Cost items	Cost (£)	Source of data
Genetic service activity		
Referral received and processed	5.07	Primary data collection
Referral triaged	1.17	Primary data collection
Request documents	8.82	Primary data collection
Clinical review of case	21.85	Primary data collection
Appointment arranged	3.49	Primary data collection
Clinic preparation	2.74	Primary data collection
Clinic appointment	104.80	Primary data collection
Post appointment letter	11.18	Primary data collection
Post appointment administration	5.17	Primary data collection
Staff salaries (London)		
Band 3, administrative assistant	17.19 per hour	NHS Agenda for change
Band 5, medical secretary	23.48 per hour	NHS Agenda for change
Band 6, administrative lead	28.74 per hour	NHS Agenda for change
Band 8, genetic counsellor	55.54 per hour	NHS Agenda for change
Registrar	61.46 per hour	PSSRU
Consultant	139.73 per hour	PSSRU
Patient management		
Mammography	45.50	(Taylor-Phillips et al. 2012)
MRI	145.88	(NHS reference costs 2012)
Mastectomy	6784.00	(NHS reference costs 2012; NICE 2013)
Salpingo-oophorectomy	3355.43	(NICE 2013; NHS reference costs 2012)

Staff salary unit costs for administrators, laboratory and clinical staff were obtained from either the NHS agenda for change or the Personal Social Services Research Unit (PSSRU) reference costs for 2013 (Unit costs of health and social care 2012). The mid-point of each grade was used and National Insurance, superannuation and overhead costs added if not already included. In addition, the cost of genetic counsellor time accounts for supervision at a ratio of 1 h consultant supervision to 12.5 h counsellor time (Torrance et al. 2006). All staff time was calculated with a London weighting of 1.19 as outlined in the PSSRU (reference costs) (Table 1) (Unit costs of health and social care 2012). The costs of clinical appointments were taken from the 2012–2013 NHS reference costs (oncology) and PSSRU costs (general practice) (Unit costs of health and social care 2012; NHS reference costs 2012).

The main (base case) analysis assumes that those affected by cancer were referred through their oncologist, and those unaffected by cancer were referred by their general practitioner. This analysis is restricted to women not within populations in whom BRCA founder mutation testing is available, such as the Ashkenazi Jewish population. Furthermore, the base case analysis assumes that all women are seen in the cancer genetic service at the age of 30 years. This age was chosen as it accommodates the years of highest relative risk of both breast and ovarian cancer in BRCA mutation carriers (Antoniou et al. 2003). All costs are presented in 2013 pounds Sterling and assumed to be incurred at the point of service delivery with the exception of surveillance which continues until the age of 70 years and hormone replacement therapy which continues until the age of 50 years. A discount rate of 3.5 % was applied to the costs associated with surveillance and hormone replacement therapy (Gray et al. 2011). This discounting rate adjusts the costs to reflect both time preference and the fact that items depreciate over time. Risk-reducing surgery is assumed to take place in the year of diagnosis and therefore not subject to discounting.

### Audit data

An audit of all clinical activity relating to breast and/or ovarian cancer patients or unaffected patients with a family history of breast and/or ovarian cancer was undertaken at the Royal Marsden Cancer Genetics Service between September 2009 and February 2010. These data were used to determine the number of patients, at first appointment, that followed each patient pathway within the audit period. Women, with breast and/or ovarian cancer, whose consultation was in relation to BRCA testing, were included; excluding those tested for founder mutations and other cancer predisposition genes and male patients. This audit excludes those that were inappropriately referred or failed to attend appointments.

### Sensitivity analysis

In order to examine how sensitive the costing results were likely to be to any assumptions that we made, sensitivity analysis was performed where some elements of resource use and costs were varied. The costs varied in the sensitivity analysis included member of clinical team involved in the patient pathway, method of consultation, cost of the test and removal of London weighting.

## Results

### Patient pathways

A total of 28 individual patient pathways were identified for the delivery of the breast and ovarian cancer genetic services, which are split into individuals affected and unaffected by cancer. A full description of all 28 pathways and their associated costs are included in the supplementary information (Supplementary Tables 1 and 2). Pathway 6 is shown in Table 2, as an exemplar, was chosen as it is the most frequent affected pathway. This pathway is for an affected individual eligible for BRCA testing, who has a negative BRCA test.

**Table 2:** Example of patient pathway units of activity, and associated costs

Pathway 6		
Activity	Sub activity	Cost (£)
Oncology referral	Oncology clinic appointment	168.00
Appointment administration	Referral received and processed	5.07
	Referral triaged	1.17
	Request documents	8.82
	Clinical review	21.85
	Appointment arranged	3.49
	Clinic preparation	2.74
Clinic related activity	Clinic appointment	104.80
	Post appointment letter	11.18
	Post appointment administration	5.17
Blood sample	Phlebotomy	3.00
BRCA full gene test		540.00
Follow up appointment administration	Appointment arranged	3.49
	Clinic preparation	2.74
Follow up clinic related activity	Clinic appointment	104.80
	Post appointment letter	11.18
	Post appointment administration	5.17
Higher risk surveillance	Mammography	628.30

The supplementary tables and Table 3 give a detailed breakdown of these pathways, but in summary, pathways 1 to 10 represent individuals referred to the cancer genetic service, who were affected with breast and/or ovarian cancer (affected patient pathways). The main differences between these 10 pathways are related to whether individuals are considered to be eligible for BRCA testing, whether they decide to undergo testing, and their subsequent management based on the test result and their family history.

**Table 3:** Pathway costs

Number	Pathway description	Pathway cost
Affected patient pathways		
1	Affected individual, BRCA mutation identified	£5606.16
2	Affected individual, known familial BRCA mutation identified	£5174.16
3	Affected individual, known familial BRCA mutation not identified	£739.88
4	Affected individual, declined BRCA testing, higher risk family history	£960.59
5	Affected individual, declined BRCA testing, moderate risk family history	£911.08
6^a^	Affected individual, BRCA testing negative, higher risk family history	£1630.96
7	Affected individual, BRCA testing negative, moderate risk family history	£1581.45
8	Affected individual, not eligible for BRCA testing, population surveillance	£501.51
9	Affected individual, not eligible for BRCA testing, moderate risk family history	£911.08
10	Affected individual, not eligible for BRCA testing, higher risk family history	£960.59
Unaffected patient pathways		
11	Unaffected individual, known familial BRCA mutation identified	£7944.56
12	Unaffected individual, known familial BRCA mutation, test declined	£835.59
13	Unaffected individual, known familial BRCA mutation not identified	£614.88
14	Unaffected individual, no testing recommended, higher risk family history	£835.59
15	Unaffected individual, no testing recommended, moderate risk family history	£786.08
16	Unaffected individual, no testing recommended, population risk	£376.51
17	Unaffected individual, affected relative eligible for BRCA testing, mutation identified in affected relative and in individual	£13,553.10
18	Unaffected individual, affected relative eligible for BRCA testing, mutation identified in affected relative but not in individual	£6223.42
19	Unaffected individual, affected relative eligible for BRCA testing, no mutation identified in affected relative, higher risk family history	£2707.29
20	Unaffected individual, affected relative eligible for BRCA testing, no mutation identified, moderate risk family history	£2608.27
21	Unaffected individual, affected relative eligible for BRCA testing, no mutation identified in relative, population risk	£1789.13
22	Unaffected individual, family eligible for BRCA testing, no relative available or relative does not get tested, higher risk family history	£835.59
23	Unaffected individual, family eligible for BRCA testing, no relative available or relative does not get tested, moderate risk family history	£786.08
24	Unaffected individual, family eligible for BRCA testing, no relative available or relative does not get tested, population risk	£376.51
25	Unaffected individual, known familial BRCA mutation, relative to be tested first, relative negative	£1118.76
26	Unaffected individual, family already tested, no mutation identified, moderate risk family history	£786.08
27	Unaffected individual, family already tested, no mutation identified, higher risk family history	£835.59
28	Unaffected individual, family already tested, no mutation identified, population risk	£376.51

^a^See Table 2 for details of this pathway

Pathways 11 to 28 represent individuals referred to cancer genetic services, who are not affected with cancer (unaffected patient pathways). The main differences between these pathways are related to whether a relative of the individual has previously undergone BRCA mutation testing, and if not, whether the individual or a relative is eligible for BRCA testing, and their subsequent management based on the test result and their family history.

### Pathway costs

Table 3 presents the total cost of each individual pathway. The most expensive pathway (£13,553.10) is that in which an unaffected individual presents, a relative of that individual has a BRCA test which is positive and the unaffected individual is also mutation positive. This reflects the management costs incurred by both individuals in the family. There are three pathways with the lowest cost (£376.51), each of which are pathways for unaffected individuals either from a family where previous testing has not identified a BRCA mutation, from a family where no relative is available for testing or from a family where no BRCA testing is recommended. In each pathway, the unaffected individual is subsequently managed at population risk. This lower cost represents the absence of genetic testing and the lower management costs.

The average cost across all 28 patient pathways in the cancer genetic services was £2227.39 per pathway, when weighted by the audit data this average was £1760.95. The average cost per pathway for a person affected with breast or ovarian cancer was £1897.75 (weighted average £2083.95) compared to £2410.53 (weighted average £1561.45) for an unaffected person. The average cost of a pathway where the presenting patient is found to carry a BRCA mutation was £8069.50, with a weighted average using audit data of £6657.08, representing higher management costs in these patients. The average cost of a pathway where a full BRCA test was carried out in a presenting patient or relative was £4462.47 (weighted average £3212.44) compared to £3118.45 (weighted average £3299.96) when a predictive BRCA test for a known mutation is performed. In the situation of BRCA testing being recommended in a relative of the presenting unaffected patient, the average cost of the pathway rose to £5376.24 (weighted average £4445.15).

### Audit data

A total of 220 women had first appointments, regarding breast and/or ovarian and BRCA testing, within the Cancer Genetics Service at the Royal Marsden NHS Foundation Trust, between September 2009 and February 2010. Of these, 84 (38 %) women were affected with breast and/or ovarian cancer, and 136 (62 %) were unaffected but concerned about their family history. A total of 72 (33 %) women were eligible for BRCA testing at the first appointment either as a full mutation screen (*n* = 42) or as a predictive test (*n* = 30).

Of the 84 women with breast and/or ovarian cancer, seen in first appointments between September 2009 and February 2010, 42 % (35/84) were eligible for, and underwent, BRCA testing. In nine patients, a mutation was identified. In the 26 where no mutation was identified 23 were eligible for higher risk surveillance and three for moderate risk surveillance. Seven women declined genetic testing, all of whom were eligible for higher risk surveillance. In 32 women, no testing was recommended; four were at population risk, 12 were eligible for moderate risk surveillance and 16 for higher risk surveillance. There were a further 10 women affected with breast and/or ovarian cancer, who had not themselves been tested, but where a familial BRCA mutation was known.

A total of 136 unaffected individuals were seen in first appointments during the audit period. In 30/136 cases, there was a known familial BRCA mutation, 22 of these underwent predictive testing; seven were found to carry a mutation. There were 35 of these 136 unaffected women in whom BRCA testing in a family member was not recommended; eight were population risk, 18 eligible for moderate risk surveillance and nine higher risk surveillance.

BRCA testing in a relative with cancer was recommended for 19 unaffected individuals. In three of these 19 cases, there was a known familial mutation in a distant relative and an intervening relative required testing prior to further management of the patient. In each of these families, the intervening relative was found not to carry a BRCA mutation, and therefore, testing was not carried out in the unaffected patient. In 16 of these 19 cases, it was recommended that an affected relative had a full BRCA test; four of these relatives were mutation positive, allowing the unaffected individual to undergo predictive testing. The period of the audit precludes inclusion of results of these predictive tests, a probability of 50 % was used to predict that two of these four unaffected individuals would be BRCA mutation carriers and two would not. There were 12 relatives found not to carry a mutation; 11 of these were families eligible for higher risk surveillance, and one was at population risk.

For 12 unaffected individuals, an affected relative had already been tested and found to be negative for BRCA mutations. Three were eligible for moderate risk surveillance and nine for higher risk surveillance. For 40 unaffected individuals, their families were potentially eligible for testing, but either no relative with cancer was available for testing, a relative was informed about eligibility but did not have testing or the unaffected individual decided not to proceed with testing. Thirty-two of these unaffected individuals were eligible for higher risk surveillance and five for moderate risk surveillance. Three cases were at population risk.

### Sensitivity

The results of the sensitivity analysis show that varying the test cost had the greatest impact on total service costs whilst varying the proportion of appointments undertaken by genetic counsellors had the least impact on overall cost of service (Table 4).

**Table 4:** Sensitivity analysis of total service cost in 6-month period

Sensitivity analysis	Cost varied	Cost of service during audit period (£)
Base case cost (100 % consultant appointments, 100 % face to face appointments, cost of test £540, London weighting)		387,409
Removal of London weighting		377,801
Varying test cost	Test at £300	375,169
	Test at £400	380,269
	Test at £600	390,469
	Test at £700	395,569
	Test at £1000	410,869
Varying proportion of consultant appointments	25 % consultant, 75 % genetic counsellor appointments	377,594
	50 % consultant, 50 % genetic counsellor appointments	380,866
	75 % consultant, 25 % genetic counsellor appointments	384,137
Varying proportion of clinic appointments	25 % telephone, 75 % clinic appointments	383,903
	50 % telephone, 50 % clinic appointments	380,396
	75 % telephone, 25 % clinic appointments	376,890

## Discussion

The detailed costing analysis presented here provides an insight into the resources required in the delivery of the current cancer genetic service for breast and ovarian cancer. The pathways address the whole model of referral to cancer genetic services including referral, stratification and management of patients; furthermore, this model of delivery is similar to services for other cancer genetic conditions such as referrals for colorectal cancer risk. Twenty-eight patient pathways were identified with associated costs ranging from £376.51 to £13,553.10 (difference of £13,176.59) depending on the testing strategy and management plan for the patient.

The burden of cost in the patient pathways presented here lies in the management of patients, in particular those identified as carrying a BRCA mutation. To fully evaluate cost-effectiveness, these data would need to be combined with outcome data, for example to include the costs saved from cancers prevented through risk-reducing surgery. The available data suggest that identifying BRCA mutations is cost-effective (Kwon et al. 2010; Holland et al. 2009; NICE 2013; Griebsch et al. 2006). Of particular relevance in the UK, the NICE guidance for familial breast cancer, using economic modelling, determined that testing affected or unaffected individuals at a risk threshold of ≥5 % was cost-effective in women under 59 years (at a CE threshold of £30,000), although the clinical guidance recommends testing for women of any age, at a risk threshold of ≥10 % (NICE 2013).

Intuitively, an unaffected individual would be expected to receive the maximum benefits of genetic testing such as a reduced incidence of primary cancers. Furthermore, in individuals found to be mutation negative, cost savings are generated from reduced surveillance (NICE 2013). From the service perspective, the most cost-efficient strategy would be to identify unaffected relatives from an affected individual in whom a BRCA mutation has been identified. These pathways are demonstrated to have an average cost of £3118.45 (£3299.96 weighted average for the RMH service). Moreover, these pathways reduce the time and expense of the ‘loops’ seen when an unaffected individual is referred to the cancer genetic service, but, though eligible, their relative with cancer has not been offered BRCA testing. In this scenario, i.e. where BRCA testing is recommended in an affected relative of an unaffected patient seen in genetics, the average pathway cost rises to £5376.24 (£4445.15 weighted average for the RMH service). Two-thirds of patients referred to the cancer genetic service were unaffected, and only one third of these were from a family where BRCA mutation testing had been performed in an affected relative.

One mechanism for reducing the time and expense of these loops would be if individuals with breast and/or ovarian cancer eligible for BRCA testing were more routinely getting access to genetic testing. Cancer genetic services have restricted capacity and would be unlikely to be able to deliver testing comprehensively to cancer patients (Fong et al. 2009). However, more access to genetic testing could be possible if testing in cancer patients became integrated into oncology services. This service model has been termed the ‘mainstreaming’ of genetic testing (Burton 2011; Burton et al. 2010; Vencken et al. 2013). The MCG programme, and other initiatives, is implementing mainstreaming for BRCA testing through oncology (www.mcgprogramme.com), in close communication with genetics. Although such initiatives typically lead to higher volumes of tests being undertaken, the resulting potential cost increases are greatly mitigated by the adoption of next-generation sequencing, which is much cheaper than Sanger sequencing. Furthermore, the identification of more affected individuals can facilitate efficient cascade testing and preventative measures potentially saving overall expenditure for the healthcare provider (NICE 2013).

Additionally, there is potential for this approach to offer two substantial advantages. Firstly, it would deliver greater equity of access to BRCA testing at guideline thresholds, and secondly, it would streamline the clinical pathways making them more time-efficient for the patient and the clinical teams, with the capacity to accommodate growing demand. It is likely that the integration of cancer genetic testing into routine patient pathways in oncology, in close liaison with genetic services, will prove to be the optimal pathway for most cancer patients. However, cost-effectiveness analysis is required to better understand the benefits that would be gained both from implementing new sequencing technologies and from broadening of testing access.

Our paper has provided a basis for understanding what resources are currently being used in cancer genetic services so that policy makers can better understand the starting point for integrating cancer gene testing into cancer care. Along with the development of mainstream testing pathways, and the harnessing of new sequencing technologies for clinical diagnostics, a comprehensive translational evidence base including service evaluation, economic evidence and careful consideration of the resource allocation challenges are essential to making genomic medicine a reality (Buchanan et al. 2013; Sullivan et al. 2012).

## Electronic supplementary material

Below is the link to the electronic supplementary material.

Supplementary Table 1 (DOCX 18 kb)

Supplementary Table 2 (DOCX 26 kb)

Supplementary Figure 1 (PDF 55 kb)

Supplementary Figure 2 (PDF 56 kb)
