# Supplementary material for: A cost analysis of a cancer genetic service model in the UK
Source: J Community Genet. 2016 Feb 27;7(3):185–94. doi: 10.1007/s12687-016-0266-4 (PMC4960025; doi:10.1007/s12687-016-0266-4)
Supplement: Supplementary file 1 — (DOCX 18 kb) [file 12687_2016_266_MOESM1_ESM.docx]

| **Pathway number** | **1** | **2** | **3** | **4** | **5** | **6** | **7** | **8** | **9** | **10** |
| --- | --- | --- | --- | --- | --- | --- | --- | --- | --- | --- |
| **Pathway description** | **Affected individual, BRCA mutation identified** | **Affected individual, known familial BRCA mutation identified** | **Affected individual, known familial BRCA mutation not identified** | **Affected individual, declined BRCA testing, higher risk family history** | **Affected individual, declined BRCA testing, moderate risk family history** | **Affected individual, BRCA testing negative, higher risk family history** | **Affected individual, BRCA testing negative, moderate risk family history** | **Affected individual, not eligible for BRCA testing, population surveillance** | **Affected individual, not eligible for BRCA testing, moderate risk family history** | **Affected individual, not eligible for BRCA testing, higher risk family history** |
| **Oncology Referral** | 168.00 | 168.00 | 168.00 | 168.00 | 168.00 | 168.00 | 168.00 | 168.00 | 168.00 | 168.00 |
| **Appointment administration** | 43.14 | 43.14 | 43.14 | 43.14 | 43.14 | 43.14 | 43.14 | 43.14 | 43.14 | 43.14 |
| **Clinic related activity** | 121.15 | 121.15 | 121.15 | 121.15 | 121.15 | 121.15 | 121.15 | 121.15 | 121.15 | 121.15 |
| **Blood sample** | 3.00 | 3.00 | 3.00 |  |  | 3.00 | 3.00 |  |  |  |
| **BRCA full gene test** | 540.00 |  |  |  |  | 540.00 | 540.00 |  |  |  |
| **BRCA predictive test** |  | 108.00 | 108.00 |  |  |  |  |  |  |  |
| **Follow up appointment administration** | 6.23 | 6.23 | 6.23 |  |  | 6.23 | 6.23 |  |  |  |
| **Follow up clinic related activity** | 121.15 | 121.15 | 121.15 |  |  | 121.15 | 121.15 |  |  |  |
| **Affected carrier management** | 4603.51 | 4603.51 |  |  |  |  |  |  |  |  |
| **Higher risk screening** |  |  |  | 628.30 |  | 628.30 |  |  |  | 628.30 |
| **Moderate risk screening** |  |  |  |  | 578.79 |  | 578.79 |  | 578.79 |  |
| **Population surveillance** |  |  | 169.22 |  |  |  |  | 169.22 |  |  |

**Supplementary Table 1**. Individuals affected with breast and/or ovarian cancer **-** patient pathways, units of activity and associated costs
