# Supplementary material for: A cost analysis of a cancer genetic service model in the UK
Source: J Community Genet. 2016 Feb 27;7(3):185–94. doi: 10.1007/s12687-016-0266-4 (PMC4960025; doi:10.1007/s12687-016-0266-4)
Supplement: Supplementary file 2 — (DOCX 26 kb) [file 12687_2016_266_MOESM2_ESM.docx]

| **Pathway number** | **11** | **12** | **13** | **14** | **15** | **16** | **17** | **18** | **19** |
| --- | --- | --- | --- | --- | --- | --- | --- | --- | --- |
| **Pathway description** | **Unaffected individual, known familial BRCA mutation identified** | **Unaffected individual, known familial BRCA mutation, test declined** | **Unaffected individual, known familial BRCA mutation not identified** | **Unaffected individual, no testing recommended, higher risk family history** | **Unaffected individual, no testing recommended, moderate risk family history** | **Unaffected individual, no testing recommended, population risk** | **Unaffected individual, affected relative eligible for BRCA testing, mutation identified in affected relative and in individual** | **Unaffected individual, affected relative eligible for BRCA testing, mutation identified in affected relative but not in individual** | **Unaffected individual, affected relative eligible for BRCA testing, no mutation identified in affected relative, higher risk family history** |
| **GP referral (patient)** | 43.00 | 43.00 | 43.00 | 43.00 | 43.00 | 43.00 | 43.00 | 43.00 | 43.00 |
| **Appointment administration (patient)** | 43.14 | 43.14 | 43.14 | 43.14 | 43.14 | 43.14 | 43.14 | 43.14 | 43.14 |
| **Clinic related activity (patient)** | 121.15 | 121.15 | 121.15 | 121.15 | 121.15 | 121.15 | 121.15 | 121.15 | 121.15 |
| **GP referral (relative)** |  |  |  |  |  |  | 43.00 | 43.00 | 43.00 |
| **Appointment administration (relative)** |  |  |  |  |  |  | 43.14 | 43.14 | 43.14 |
| **Clinic related activity (relative)** |  |  |  |  |  |  | 121.15 | 121.15 | 121.15 |
| **Blood sample (relative)** |  |  |  |  |  |  | 3.00 | 3.00 | 3.00 |
| **BRCA full gene test (relative)** |  |  |  |  |  |  | 540.00 | 540.00 | 540.00 |
| **BRCA predictive test (relative)** |  |  |  |  |  |  |  |  |  |
| **Follow up appointment administration (relative)** |  |  |  |  |  |  | 6.23 | 6.23 | 6.23 |
| **Follow up clinic related activity (relative)** |  |  |  |  |  |  | 121.15 | 121.15 | 121.15 |
| **Affected carrier management (relative)** |  |  |  |  |  |  | 4603.51 | 4603.51 |  |
| **Population surveillance (relative)** |  |  |  |  |  |  |  |  |  |
| **Moderate risk screening (relative)** |  |  |  |  |  |  |  |  |  |
| **Higher risk screening (relative)** |  |  |  |  |  |  |  |  | 628.30 |
| **Follow up appointment administration (patient)** |  |  |  |  |  |  | 6.23 | 6.23 | 6.23 |
| **Follow up clinic related activity (patient)** |  |  |  |  |  |  | 121.15 | 121.15 | 121.15 |
| **Blood sample (patient)** | 3.00 |  | 3.00 |  |  |  | 3.00 | 3.00 | 3.00 |
| **BRCA predictive test (patient)** | 108.00 |  | 108.00 |  |  |  | 108.00 | 108.00 | 108.00 |
| **Follow up appointment administration (patient)** | 6.23 |  | 6.23 |  |  |  | 6.23 | 6.23 | 6.23 |
| **Follow up clinic related activity (patient)** | 121.15 |  | 121.15 |  |  |  | 121.15 | 121.15 | 121.15 |
| **Unaffected carrier management (patient)** | 7498.90 |  |  |  |  |  | 7498.90 |  |  |
| **Population surveillance (patient)** |  |  | 169.22 |  |  | 169.22 |  | 169.22 |  |
| **Moderate risk screening (patient)** |  |  |  |  | 578.79 |  |  |  |  |
| **Higher risk screening (patient)** |  | 628.30 |  | 628.30 |  |  |  |  | 628.30 |

| **Pathway number** | **20** | **21** | **22** | **23** | **24** | **25** | **26** | **27** | **28** |
| --- | --- | --- | --- | --- | --- | --- | --- | --- | --- |
| **Pathway description** | **Unaffected individual, affected relative eligible for BRCA testing, no mutation identified in affected relative, moderate risk family history** | **Unaffected individual, affected relative eligible for BRCA testing, no mutation identified in affected relative, population risk** | **Unaffected individual, family eligible for BRCA testing, no relative available or relative does not get tested, higher risk family history** | **Unaffected individual, family eligible for BRCA testing, no relative available or relative does not get tested, moderate risk family history** | **Unaffected individual, family eligible for BRCA testing, no relative available or relative does not get tested, population risk** | **Unaffected individual, known familial BRCA mutation, relative to be tested first, relative negative** | **Unaffected individual, family already tested, no mutation identified, moderate risk family history** | **Unaffected individual, family already tested, no mutation identified, higher risk family history** | **Unaffected individual, family already tested, no mutation identified, population risk** |
| **GP referral (patient)** | 43.00 | 43.00 | 43.00 | 43.00 | 43.00 | 43.00 | 43.00 | 43.00 | 43.00 |
| **Appointment administration (patient)** | 43.14 | 43.14 | 43.14 | 43.14 | 43.14 | 43.14 | 43.14 | 43.14 | 43.14 |
| **Clinic related activity (patient)** | 121.15 | 121.15 | 121.15 | 121.15 | 121.15 | 121.15 | 121.15 | 121.15 | 121.15 |
| **GP referral (relative)** | 43.00 | 43.00 |  |  |  | 43.00 |  |  |  |
| **Appointment administration (relative)** | 43.14 | 43.14 |  |  |  | 43.14 |  |  |  |
| **Clinic related activity (relative)** | 121.15 | 121.15 |  |  |  | 121.15 |  |  |  |
| **Blood sample (relative)** | 3.00 | 3.00 |  |  |  | 3.00 |  |  |  |
| **BRCA full gene test (relative)** | 540.00 | 540.00 |  |  |  |  |  |  |  |
| **BRCA predictive test (relative)** |  |  |  |  |  | 108.00 |  |  |  |
| **Follow up appointment administration (relative)** | 6.23 | 6.23 |  |  |  | 6.23 |  |  |  |
| **Follow up clinic related activity (relative)** | 121.15 | 121.15 |  |  |  | 121.15 |  |  |  |
| **Affected carrier management (relative)** |  |  |  |  |  |  |  |  |  |
| **Population surveillance (relative)** |  | 169.22 |  |  |  | 169.22 |  |  |  |
| **Moderate risk screening (relative)** | 578.79 |  |  |  |  |  |  |  |  |
| **Higher risk screening (relative)** |  |  |  |  |  |  |  |  |  |
| **Follow up appointment administration (patient)** | 6.23 | 6.23 |  |  |  | 6.23 |  |  |  |
| **Follow up clinic related activity (patient)** | 121.15 | 121.15 |  |  |  | 121.15 |  |  |  |
| **Blood sample (patient)** | 3.00 | 3.00 |  |  |  |  |  |  |  |
| **BRCA predictive test (patient)** | 108.00 | 108.00 |  |  |  |  |  |  |  |
| **Follow up appointment administration (patient)** | 6.23 | 6.23 |  |  |  |  |  |  |  |
| **Follow up clinic related activity (patient)** | 121.15 | 121.15 |  |  |  |  |  |  |  |
| **Unaffected carrier management (patient)** |  |  |  |  |  |  |  |  |  |
| **Population surveillance (patient)** |  | 169.22 |  |  | 169.22 | 169.22 |  |  | 169.22 |
| **Moderate risk screening (patient)** | 578.79 |  |  | 578.79 |  |  | 578.79 |  |  |
| **Higher risk screening (patient)** |  |  | 628.30 |  |  |  |  | 628.30 |  |

**Supplementary Table 2.** Unaffected individuals – patient pathways, units of activity and associated costs
