# Supplementary material for: A cost analysis of a cancer genetic service model in the UK
Source: J Community Genet. 2016 Feb 27;7(3):185–94. doi: 10.1007/s12687-016-0266-4 (PMC4960025; doi:10.1007/s12687-016-0266-4)
Supplement: Supplementary file 3 — (PDF 55 kb) [file 12687_2016_266_MOESM3_ESM.pdf]

Protocol 2
BRCA1 and *BRCA2* mutation testing guidelines

A
Woman with **breast cancer** who →

1) has bilateral BC and both cancers diagnosed < 50 yrs
2) has triple negative BC diagnosed < 50 yrs
3) has OC
4) has bilateral BC and a relative with BC < 60 yrs
5) has a relative with BC and both diagnosed < 45 yrs
6) has relatives with cancer and a Manchester Score ≥15

B
Woman with **ovarian cancer** who →

1) has BC
2) has a relative with OC or MBC
3) has relatives with cancer and a Manchester Score ≥ 15

C
Male with **breast cancer** who →

1) has a relative with OC or MBC
2) has relatives with cancer and a Manchester Score ≥ 15

D
Individual **unaffected** with cancer who →

1) meets unaffected testing criteria *

Results
***BRCA1/2* mutation identified:** see Protocol 3
***BRCA1/2* mutation not identified:** see Protocol 1 for breast screening recommendation
***BRCA1/2* variants/VUS identified:** see Protocol 4 and email: vus@icr.ac.uk

Notes*
The above criteria relate to a full gene screen of *BRCA1* and *BRCA2*, individuals of Ashkenazi Jewish heritage may be eligible for founder mutation testing.*

Key
Relative = first degree or second degree relative only, except when calculating a Manchester score*. Female relatives through an intervening male shift up one degree of relationship*
BC = breast cancer
MBC = male breast cancer
OC = ovarian cancer
Triple negative breast cancer = breast tumour negative for oestrogen receptor (ER), progesterone receptor (PR) and HER2 expression

Manchester Score*

Cancer, age at diagnosis	Score
♀ Breast Cancer, <30	11
♀ Breast Cancer, 30-39	8
♀ Breast Cancer, 40-49	6
♀ Breast Cancer, 50-59	4
♀ Breast Cancer, > 59	2
♂ Breast Cancer, <60	13
♂ Breast Cancer, > 59	10
Ovarian Cancer, <60	13
Ovarian Cancer, >59	10
Pancreatic Cancer	1
Prostate Cancer, <60	2
Prostate Cancer, >59	1

** See FAQ document. http://www.icr.ac.uk/protocols*

01/01/2013
