# Supplementary material for: A cost analysis of a cancer genetic service model in the UK
Source: J Community Genet. 2016 Feb 27;7(3):185–94. doi: 10.1007/s12687-016-0266-4 (PMC4960025; doi:10.1007/s12687-016-0266-4)
Supplement: Supplementary file 4 — (PDF 56 kb) [file 12687_2016_266_MOESM4_ESM.pdf]

Protocol 1

Mammographic screening guidelines for women With a family history of breast and/or ovarian cancer

Key
FDR = First degree relative
SDR = Second degree relative
TDR =Third degree relative
BC = Breast Cancer
OC = Ovarian Cancer

Category B1 screening
Annual mammography 40-50 years
Three-yearly mammography 50-70 years

Category B2 screening
Annual mammography 40-50 years
18-monthly mammography 50-60 years
Three-yearly mammography 60-70 years

**1 relative with breast cancer**[1]	One female FDR with BC < 40 yrs	
	One male FDR with BC at any age	
	One FDR with bilateral BC at any age	Both cancers < 50 yrs
**1 relative with breast and ovarian cancer**[2]	One FDR with BC and OC at any age	BC < 50 yrs
**2 relatives with breast cancer**[1]	Two female FDR with BC at any age	Both < 50 yrs or One < 35 yrs
	One female FDR and one female SDR with BC at any age	Both < 40 yrs or FDR < 35 yrs
	Bilateral BC any age and another relative < 60 yrs (at least one must be FDR)	At least two cancers < 55 yrs
	One male BC and one female BC at any age (at least one must be FDR)	Female BC < 50 yrs
**2 relatives: one with breast and one with ovarian cancer**[2]	One OC and one BC at any age (at least one must be FDR)	BC < 50 yrs
**2 or more relatives with ovarian cancer**[2]		Two OC any age (at least one must be FDR)
**3 relatives with breast cancer**[1]	Three female FDR or SDR with BC at any age	Two are FDR or Two < 55 yrs and one is FDR or FDR < 35 yrs
	Three FDR or SDR with BC at any age at least one must be male BC	Two are MBC or Two are female BC < 60 yrs (at least one must be FDR)
	Three FDR or SDR with BC at any age at least one must be bilateral BC	One relative is a FDR
**3 relatives with breast or ovarian cancer**[2]	Three FDR or SDR with BC or OC at any age	All BC < 60 yrs or At least two are OC (at least one must be FDR
**4 or more relatives with breast or ovarian cancer**[2]	FDR/SDR/TDR with BC or OC at any age (at least one must be SDR)	One relative is a FDR

Notes*

- If the consultee is affected with breast cancer and has residual breast tissue, count as a FDR.
- An affected female SDR through a male FDR is equivalent to a FDR.
- One relative must be a FDR of the consultee, unless otherwise specified.

BRCA1/2* negative families

[1] For breast only families, a negative *BRCA1/2* test does not alter the mammographic screening category.

[2] For breast-ovarian families, a negative *BRCA1/2* test, may alter mammographic screening category. The screening category should be recalculated on the basis of the breast cancers in the family alone.

* *See FAQ document. http://www.icr.ac.uk/protocols*
